# Cardiomyocyte uptake mechanism of a hydroxyapatite nanoparticle mediated gene delivery system

**DOI:** 10.3762/bjnano.11.150

**Published:** 2020-11-05

**Authors:** Hiroaki Komuro, Masahiro Yamazoe, Kosuke Nozaki, Akiko Nagai, Tetsuo Sasano

**Affiliations:** 1 Department of Cardiovascular Physiology, Graduate School of Medical and Dental Sciences, Tokyo Medical and Dental University, Bunkyo, Tokyo 113-8510, Japan; 2 Department of Cardiovascular Medicine, Graduate School of Medical and Dental Sciences, Tokyo Medical and Dental University, Bunkyo, Tokyo 113-8510, Japan; 3 Department of Bio-informational Pharmacology, Medical Research Institute, Tokyo Medical and Dental University, Bunkyo, Tokyo 113-8510, Japan; 4 Department of Restorative Sciences, Graduate School of Medical and Dental Sciences, Tokyo Medical and Dental University, Bunkyo, Tokyo 113-8510, Japan; 5 Department of Anatomy, School of Dentistry, Aichi Gakuin University, Chikusa, Nagoya 464-8650, Japan

**Keywords:** cardiomyocyte, endocytosis, gene delivery system, hydroxyapatite nanoparticles, macropinocytosis

## Abstract

Gene therapy has been explored as a future alternative for treating heart disease. Among several gene delivery systems aimed at penetrating specific target cells, we focused on safe and non-viral gene delivery materials with a high transfection efficiency. Although various techniques have been developed, the mechanisms underlying the cellular uptake of gene delivery materials have not yet been sufficiently studied in cardiomyocytes. The aim of this study was to determine how hydroxyapatite (HAp) nanoparticles contribute to the delivery of plasmid DNA (pDNA) into cardiomyocytes. We fabricated HAp nanoparticles using the water-in-oil (W/O) emulsion method and used these nanoparticles as the delivery vector for transfecting cardiomyocyte-derived HL-1 cells. HAp exhibited particles on the nanoscale and with a low cytotoxicity in HL-1 cells. The transfection assay performed with several endocytosis inhibitors suggested that the HAp/pDNA complexes were internalized by HL-1 cells through macropinocytosis. Furthermore, this HL-1 cell uptake was generated in response to HAp stimulation. Thus, HAp is a positive regulator of macropinocytosis in HL-1 cells and a good system for gene delivery in cardiomyocytes.

## Introduction

Heart disease is one of the major causes of death and accounts for approximately one in every three deaths worldwide every year [1]. Since cardiomyocytes have very limited regenerative functions, the heart becomes fibrotic after the cardiomyocytes become damaged during certain events such as myocardial infarction, leading to a decline in the contraction function and eventually heart failure. Patients with advanced heart failure only have the option of a heart transplant from limited donor organs, prompting the need for alternative treatment strategies. Recently, there has been significant interest in gene therapy, which is an experimental technique that uses specific targeting genes to treat or prevent diseases [2]. Since gene therapy for heart disease will potentially become the standard treatment in the future, many studies using viral and non-viral vectors have been conducted in cardiomyocytes. However, most of these studies still use adeno-associated viral vectors which can be a potential infection risk. Therefore, safety measures should always be considered during their usage [3]. Recently, a nanotechnology-based non-viral vector system with the potential to overcome many limitations regarding safety has attracted significant attention [4–5]. The calcium phosphate (CaP) co-precipitation method has been extensively used for gene delivery due to its excellent biocompatibility and simple preparation [6]. CaP is commonly considered as one of the most important inorganic materials for medical and dental applications, such as dental implants, orthopedics, and drug delivery systems, since it has similar elements found in bone and teeth. In addition, CaP stabilizes the nucleic acid against nuclease degradation, forms ionic interactions with the phosphates of DNA, and its biodegradation is pH-sensitive [7–9]. Besides, CaP can be internalized in targeting cells though the endocytic pathway. Later on, CaP is dissolved in the endosome under acidic conditions, which contributes to the DNA release into the cytosol before the endosome–lysosome fusion. Although there are a number of advantages in using CaP for gene delivery, the transfection efficiency of CaP/DNA is relatively low according to various preparation parameters [10]. In particular, particle aggregation needs to be improved in order to reduce cellular uptake through the endocytic pathway. Hydroxyapatite (HAp, Ca_10_(PO_4_)_6_(OH)_2_) is one of the most stable forms of CaP, and diverse methods for preparing HAp nanoparticles have been reported. Among these methods, the microemulsion method has the advantage of controlling the spherical-like morphology and the size of nanoparticles by using a surfactant solution [11–12]. Therefore, this method may provide a solution to the current problem of using calcium phosphate. The cellular uptake performance is important for a successful vector-mediated gene transfection. In the cellular uptake process, the internalization pathway is an essential factor to prevent the fate of lysosomal degradation by facilitating the release of the gene into the cytoplasm. Endocytosis has been well known as the main mechanism for cellular uptake of nanoparticles into mammalian cells [13–14]. The endocytosis process encompasses four main routes [15]: phagocytosis, clathrin-mediated endocytosis, caveolae-mediated endocytosis, and micropinocytosis. These routes depend heavily on the cell type studied [16–17] and on the vector properties, such as size, shape, chemical composition, and surface chemistry [18–19]. Therefore, the fate of the vector/DNA complex taken up by a cell depends on the type of endocytosis involved in the uptake process [20–21]. Therefore, understanding the cellular uptake mechanism of HAp nanoparticles may be useful for the design of more efficient gene-delivery vectors. We previously demonstrated that the cellular uptake of HAp nanoparticles into endothelial cells (ECs) was mainly through caveolae-mediated endocytosis [22]. In this study, we show that HAp nanoparticles are vectors that effectively transfect cardiomyocytes in comparison to ECs and the uptake of HAp in cardiomyocytes is through macropinocytosis. Furthermore, this pathway can be activated by the stimulation of HAp. The HL-1 cells were used as the in vitro model of cardiomyocytes in this study [23]. Since HL-1 cells are derived from mouse atrial myocytes they have an adult cardiomyocyte phenotype with the expression of cardiac-specific functional receptors.

## Results

### Characterization of HAp nanoparticles

**Figure 1:**
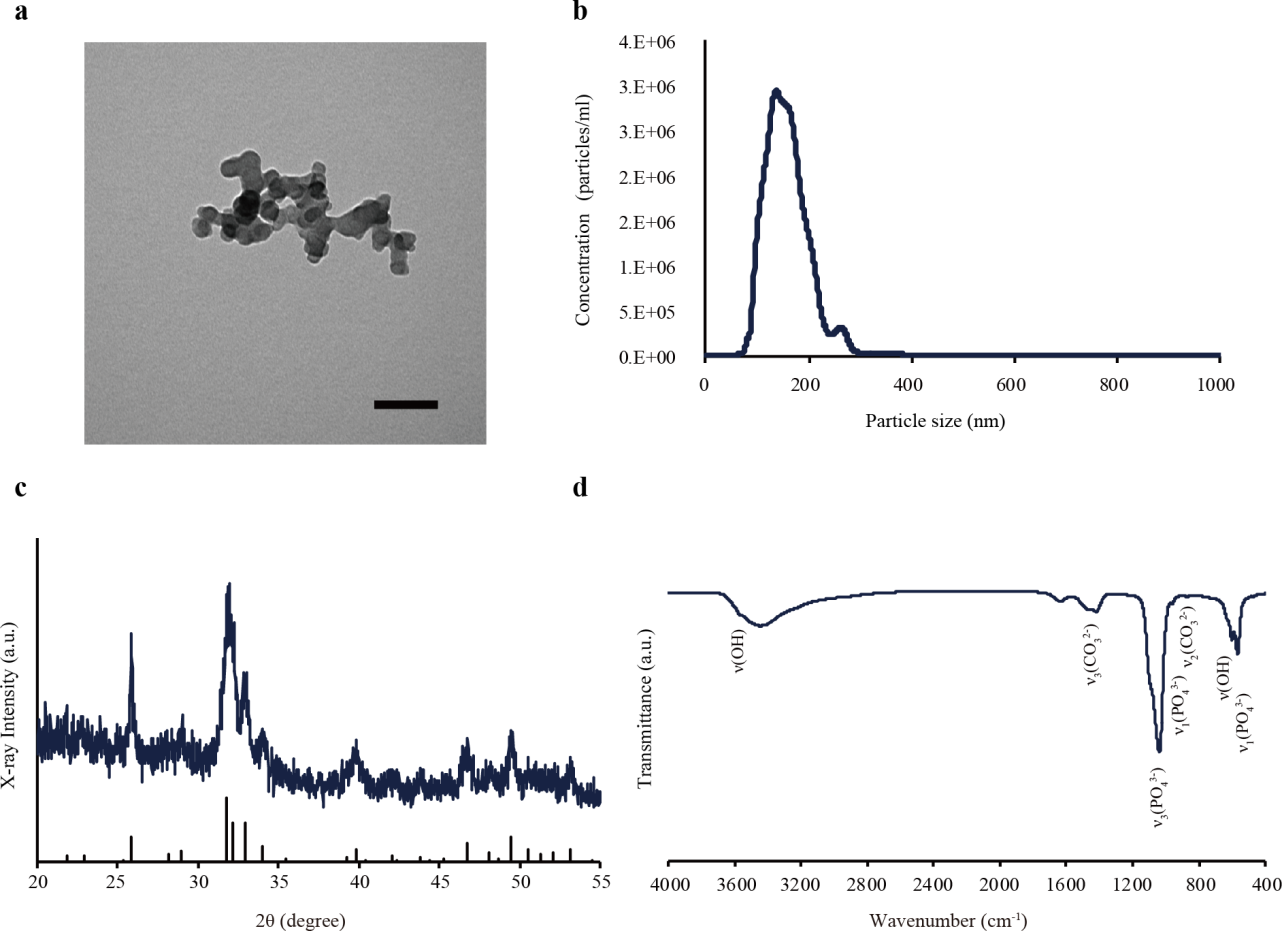
Characterization of the prepared HAp nanoparticles using the W/O emulsion method. (a) A representative TEM image of HAp nanoparticles. The nanoparticles exhibited uniform size and spherical-like morphology. Scale bar: 100 nm. (b) The size distribution of HAp nanoparticles was determined using nanoparticle tracking analysis (NTA). The mean diameter was 159 ± 47 nm. (c) An X-ray diffractogram of the HAp nanoparticles in the range of 2θ = 20–60°. The spectra revealed the characteristic peaks of HAp according to ICDD no. 09-0432 (vertical lines). (d) The FTIR spectra of the HAp nanoparticles in the range of 4000–400 cm^−1^. The characteristic peaks of HAp are indicated.

The HAp nanoparticles were prepared using the water-in-oil (W/O) emulsion method. The characterization of the prepared HAp nanoparticles was carried out using transmission electron microscopy (TEM), X-ray diffraction (XRD), and Fourier-transform infrared spectroscopy (FTIR). TEM provided insight into the morphology of the obtained product. As presented in Figure 1a, the nanometer-sized particles exhibited identical spherical-like morphology. The average particle diameter of the prepared HAp was 159 ± 47 nm (Figure 1b). The XRD pattern of the product is illustrated in Figure 1c. All the diffraction peaks of the as-prepared sample are consistent with the characteristic peaks of the standard hydroxyapatite peak positions and with the corresponding intensities of the diffraction peaks for HAp (International Centre for Diffraction Data, ICDD, no. 09-0432, vertical lines). The FTIR absorption spectra of the specimens are shown in Figure 1d. The characteristic peaks for PO_4_^3−^ appeared at approximately 1040, 960, and 560 cm^−1^. The broad bands at 3410 and 1615 cm^−1^ correspond to the bending modes of the hydroxyl group in the absorbed water, while the peaks at 3570 and 620 cm^−1^ are attributed to the stretching and bending modes, respectively, of the hydroxyl group in the HAp crystalline structure. Furthermore, the carbonate anion (CO_3_^2−^) group adsorption bands were detected near 860 and 1420 cm^−1^. During the wet process, the HAp preparations can become easily contaminated with CO_3_^2−^ emanating from atmospheric CO_2_. This material was found to correspond to the B-type carbonate-containing HAp in which the phosphate group was substituted by the carbonate group absorption band in the FTIR spectrum. The carbonate amount, including the HAp crystalline structure that was calculated in a previous report, was approximately 1.3 wt % [24].

### Cytotoxicity assay

**Figure 2:**
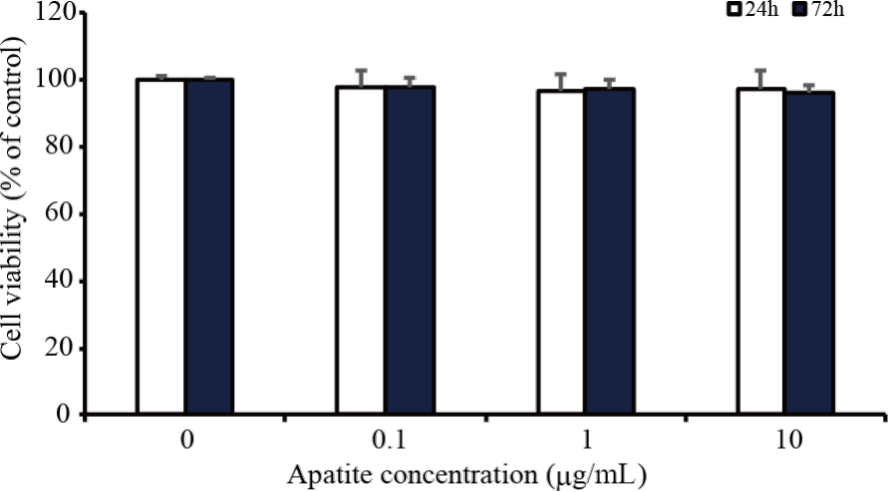
MTT assay of HL-1 cells treated with HAp nanoparticles for 24 (white bars) and 72 h (black bars) was used to determine cytotoxicity. Non-treated cells were used as the control. Values are presented as the mean ± SD (*n* = 4).

Dose-dependent cytotoxicity of HAp/pDNA complexes on HL-1 cells was investigated in the concentration range of 0.1–10 µg/mL. The 3-(4,5-dimethylhiazol-2-yl)-2,5-diphenyl-2*H*-tetrazolium bromide (MTT) assay was used to assess cytotoxicity. No differences in cell viability were observed among the three concentrations of HAp/pDNA complexes used at 24 and 72 h (Figure 2). The results suggested that HAp exhibits little cytotoxicity within the concentration range used in this study.

### Transfection efficiency

**Figure 3:**
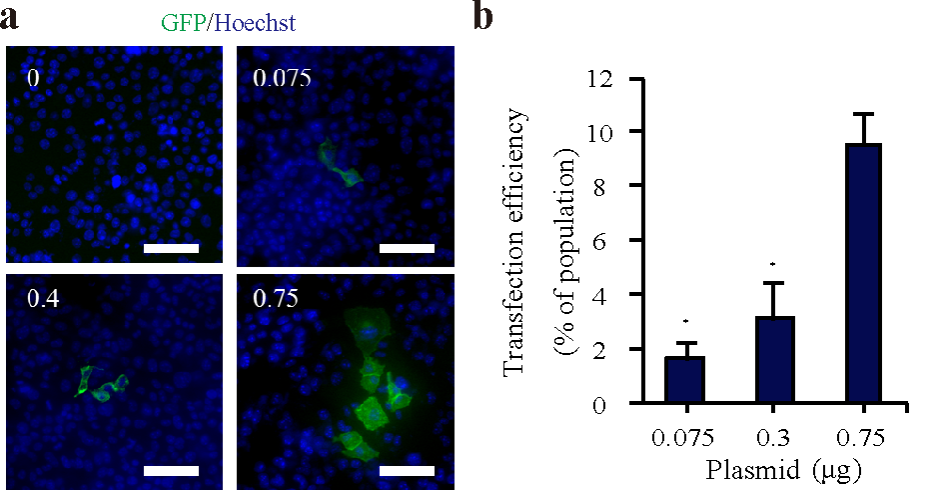
EGFP gene transfection into HL-1 cells using HAp nanoparticles. (a) Representative fluorescence images of HL-1 cells treated with HAp/pEGFP complexes. The nuclei were stained using Hoechst stain (blue). Scale bar: 100 µm. (b) The transfection efficiency of the complexes 24 h post-transfection. Values are presented as the mean ± SD (*n* = 4). *p < 0.05.

To test the gene transfection potential of the HAp nanoparticle vector in HL-1 cells, we used plasmid-enhanced green fluorescent protein (pEGFP) as a model plasmid and evaluated the transfection efficiency via fluorescence microscopy. First, we used HAp (1 µg/mL) mixed with 0.075, 0.30, and 0.75 µg pEGFP, based on our previous results with endothelial cells. Fluorescence microscopy images showed that the highest transfection efficiency was observed with 0.75 µg of pEGFP (Figure 3). No gene expression was obtained by adding only pEGFP in HL-1 cells (data not shown). The results shown in Figure 3b demonstrated that the transfection efficiency of pEGFP increased in a dose-dependent manner. The amount of 0.75 µg was selected for subsequent cell experiments. The transfection efficiency of the HAp vector in HL-1 cells was three times higher than that of the endothelial cells in our previous study [22].

### Endocytic pathway

**Figure 4:**
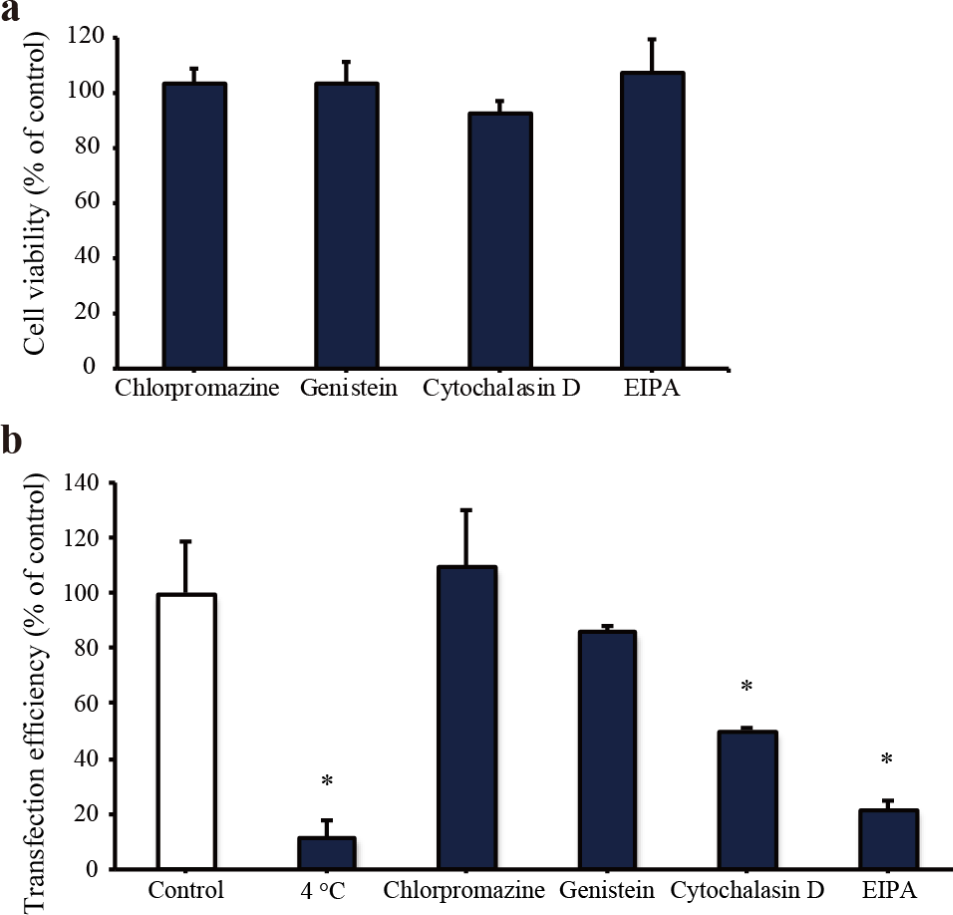
(a) Cytotoxicity assay of HL-1 cells incubated with endocytosis inhibitors for 24 h. (b) Effects of the endocytosis inhibitors on the transfection efficiency of HAp/pEGFP complexes into HL-1 cells. Values are presented as the mean ± SD (*n* = 4). *p < 0.05.

Under physiological conditions, nanoparticles can be taken up by cells via passive transport or active transport. Most nanoparticles are taken up by endocytosis (i.e., active transport) and rarely by direct penetration through the plasma membrane (i.e., passive transport). The endocytic pathway is an energy-dependent process; therefore, it can be prevented by lowering the incubation temperature to 4 °C. First, to determine whether the incorporation of HAp nanoparticles into HL-1 cells is a passive or an active transport, we examined the transfection efficiency of HAp/pEGFP complexes at 4 and 37 °C (control). The results were expressed as percentages relative to the control value. The transfection efficiency in HL-1 cells was significantly reduced at 4 °C in comparison to that at 37 °C, suggesting that the cellular uptake of HAp/pDNA complexes in HL-1 cells is primarily through an energy-dependent process. Next, we investigated endocytic pathways associated with the HAp nanoparticle in HL-1 cells using specific pharmacological inhibitors. Endocytic pathways are divided into clathrin-mediated endocytosis, caveolae-mediated endocytosis, and macropinocytosis. The pathways were analyzed by measuring the effect of the endocytosis inhibitors on the transfection efficiency. The inhibitors used were chlorpromazine, for clathrin-mediated endocytosis; genistein, for caveolae-mediated endocytosis; cytochalasin D and the amiloride derivative 5-(*N*-ethyl-*N*-isopropyl) amiloride (EIPA), for macropinocytosis. These inhibitors, at the concentration used in this study, did not generate cytotoxicity in HL-1 cells when added 24 h prior to the experiments (Figure 4a). As shown in Figure 4b, both chlorpromazine and genistein did not prevent cellular uptake when compared to non-treated cells. These results indicated that clathrin-mediated endocytosis and caveolae-mediated endocytosis were not involved in the internalization of HAp by HL-1 cells. However, the cellular uptake efficiency of HAp was reduced by 50% and 79% upon cytochalasin D and EIPA treatment, respectively, when compared to non-treated cells. These results revealed the importance of macropinocytosis for the cellular uptake of HAp into HL-1 cells.

### Macropinocytosis activity assay

**Figure 5:**
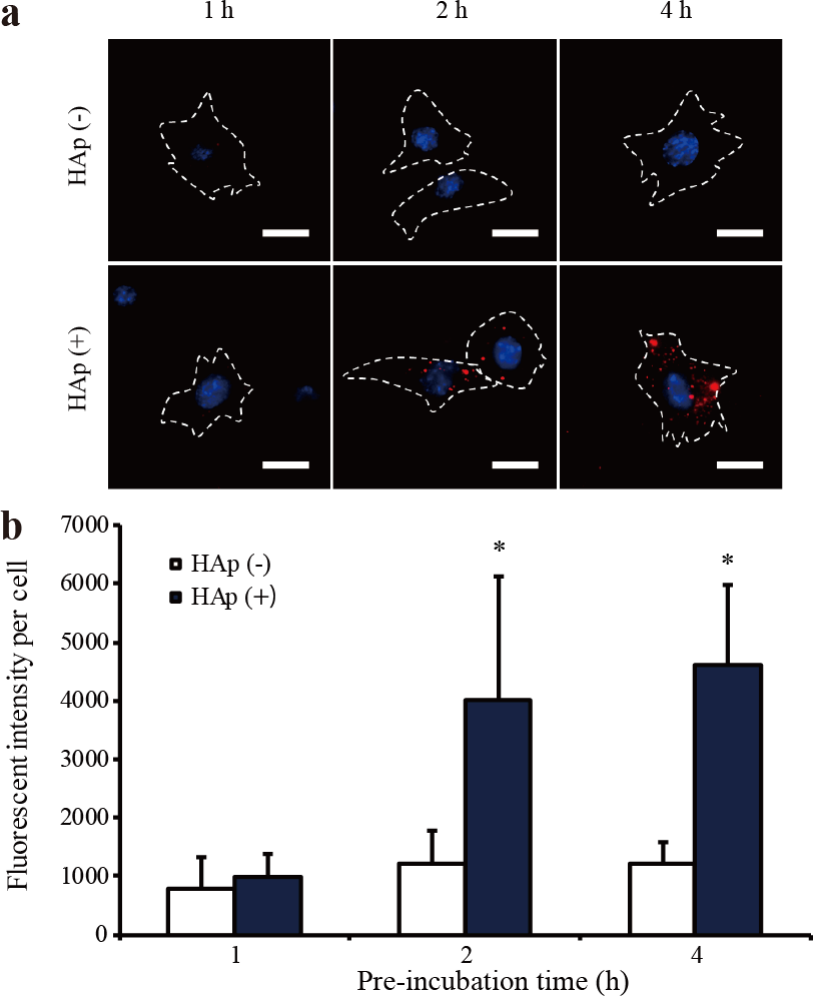
Macropinocytosis activity induced by HAp nanoparticles (1 µg/mL) in HL-1 cells. (a) HL-1 cells were pre-incubated in the presence or absence of HAp for 1, 2, and 4 h, and then treated with TMR–dextran for 30 min. Nuclei were stained using Hoechst stain (blue). Scale bar: 10 µm. (b) Quantification of fluorescence intensity per cell number. Values are presented as the mean ± SD (*n* = 25). *p < 0.05.

To further understand the mechanism of gene delivery into HL-1 cells using HAp, we investigated the HAp-induced macropinocytosis activity using neutral tetramethylrhodamine-conjugated dextran (TMR–dextran). TMR–dextran is commonly used as an indicator of the macropinocytic pathway [25], enabling the quantification of macropinocytosis. The quantification of TMR–dextran was performed by measuring the fluorescence intensity per cell, using fluorescence microscopy images, in accordance with a previous report. As shown in Figure 5a, more red spots corresponding to TMR–dextran fluorescence signals were observed in HAp-treated cells in comparison to non-treated cells. Quantitative analysis revealed that the increase in macropinocytosis activity depends on the pretreatment time: The macropinocytosis activity following a 4 h-HAp-treatment was observed to be four times higher than in the corresponding non-treated group (Figure 5b). An enhanced activity was not observed when cytochalasin D and EIPA were added together. These results imply that the gene delivery vector HAp effectively enhances macropinocytosis in HL-1 cells.

## Discussion

The development of gene therapy is essential for generating new treatment options for cardiovascular disease. We focused on safe non-viral vectors using nanotechnology. Although nanoparticle-based gene-transfection methods have been proposed for gene delivery into target cells and tissues, there are still many limitations, such as low transfection efficiency and reproducibility, that must be overcome for these methods to be successfully used in clinical applications. We were able to successfully produce HAp nanoparticles with less aggregation using the W/O emulsion method, as shown in a previous study. HAp nanoparticles have been extensively investigated in various biomedical applications. However, the cellular uptake mechanism of HAp nanoparticles into cardiomyocytes remains unknown. In the present study, we focused on verifying whether the cardiomyocyte cell line HL-1 was able to internalize HAp nanoparticles and the mechanism through which these cells performed the internalization. First, the results from transfection efficiency experiments indicated that HAp nanoparticles had a higher gene transfection efficiency in cardiomyocytes than they had in ECs. As shown in previous reports, the transfection efficiency was different for each cell type studied [17,26–27]. Next, we explored the cellular uptake mechanism of HAp nanoparticles into HL-1 cells. In ECs, HAp nanoparticles were internalized via caveolae-mediated endocytosis. Endocytosis experiments showed that both cytochalasin D and EIPA inhibited the endocytosis of HL-1 cells, whereas chlorpromazine and genistein had no significant inhibiting effects. These results implied that the uptake of HAp nanoparticles into HL-1 cells did not occur via clathrin and caveolae-mediated endocytosis, but rather via macropinocytosis. The physiological mechanism of macropinocytosis has been recently elucidated [28–29]. In previous reports, macropinosomes have been considered to be inherently leaky vesicles comparable to other types of endosomes [21,30–32]. In this study, since lysosomal degradation of pDNA was low, the macropinocytosis pathway could be highlighted as one of the factors that contributed to the increase in the transfection efficiency in HL1 cells compared to that in ECs. The cellular uptake through macropinocytosis can be efficiently induced by pretreating the cells with physiologically active substances, such as the epidermal growth factor (EGF), phorbol myristate acetate (PMA), and fetuin [32–34]. To elucidate the ability of HAp nanoparticles to actively induce macropinocytosis in HL-1 cells, we measured the number of TMR–dextran particles internalized by HL-1 cells following the HAp pretreatment. Large dextran particles are generally used as a macropinocytosis marker since their uptake is mediated through micropinocytosis and not through clathrin- or caveolae-mediated endocytosis [35]. After incubating the cells with TMR–dextran for 30 min, the number of internalized TMR–dextran particles was higher in the presence than in the absence of HAp (Figure 5). Extracellular calcium ions are proposed to act as a stimulant for triggering macropinocytosis in macrophages and neurons [25,36]. The extracellular calcium-sensing receptor (CaSR) is expressed in cardiomyocytes as well as in various other cells [37]. The results suggested that calcium ions from HAp particles might activate macropinocytosis in HL-1 cells. CaSR has different functions depending on the cell type. In osteoclasts, for example, it directs migration toward bone tissue for bone remodeling, whereas in macrophages it aids in the antigen acquisition at inflammation sites. Further studies regarding the role of CaSR and its downstream signaling pathways in HL-1 cells are required. Although the HAp-based vector used here has a low transfection efficiency, it has already been used as a gene delivery system in in vivo cancer research experiments [38]. Furthermore, this system might be useful for increasing the knowledge about cellular uptake mechanisms in heart cells, which are difficult to transfect.

## Conclusion

In summary, we developed HAp nanoparticles as a non-viral vector system for gene transfection in cardiomyocytes. The HAp/pDNA complex exhibited enhanced cellular uptake via the macropinocytosis pathway. Moreover, the macropinocytosis uptake was induced by HAp. To the best of our knowledge, this is the first report examining macropinocytosis as the uptake mechanism of HAp nanoparticles in HL-1 cells. Therefore, we proposed the usage of the HAp vector to transfect cardiomyocytes since it is safe for the cells and can trigger macropinocytosis.

## Experimental

### Preparation of HAp nanoparticles

HAp nanoparticles were prepared using the W/O emulsion method, as described in our previous study. The starting materials included 80 mL of dodecane (CH_3_(CH_2_)_10_CH_3_) in the oil phase, 1.0 g of pentaethylene glycol dodecyl ether (C_22_H_46_O_6_) as a non-ionic surfactant, and 2.5 M of calcium hydroxide. After stirring these reagents for 1 h at 50 °C, 1.25 M of potassium dihydrogen phosphate was added to these solutions. After 24 h, the product was centrifuged and washed with distilled water and ethanol to remove oil and surfactant. The resulting particles were dispersed in distilled water. Physicochemical characteristics were examined using XRD (D8 Advance, Bruker), FTIR (FT/IR-4100, Jasco), and TEM (H-7100, Hitachi). The product was filtered using a 0.45 µm filter to remove aggregated nanoparticles. The concentration of the filtered solutions was measured using a calcium assay kit in accordance with the guidelines of the manufacturer (BioAssay Systems). TEM was performed to observe the size and shape of HAp nanoparticles at an acceleration voltage of 75 kV. The particle size distribution of the HAp nanoparticles was measured via NTA (NanoSight NS10, Malvern).

### Cell culture

HL-1 is a cell line derived from mouse atrial myocytes, which was originally isolated and characterized by Dr. Claycomb (University of Louisiana) [39]. HL-1 cells (murine cardiomyocytes) were seeded and grown in Claycomb culture medium (Sigma) supplemented with 10% of fetal bovine serum (Sigma), 0.1 mM of norepinephrine (Sigma), 2 mM of ʟ-glutamine (Wako), and 1% of penicillin/streptomycin (Gibco) as previously published [39]. All cell culture dishes and plates were coated with a 25 μg/mL fibronectin solution (Wako) prepared in a 0.02% gelatin solution (Wako) in order to increase cell adhesion. The cells were grown in a humidified incubator at 37 °C containing 5% CO_2_.

### Cytotoxicity assay

The cytotoxicity of HAp/pDNA complexes or endocytosis inhibitors was verified by an MTT colorimetric assay (Dojindo). HL-1 cells were seeded in 96-well plates at a density of 1.0 × 10^4^ cells/well. After culturing HL-1 cells for 24 h, HAp nanoparticles (at concentration values of 0, 0.1, 1, and 10 µg/mL) were mixed with 0.75 µg of pEGFP or with endocytosis inhibitors (10 μM of chlorpromazine, 20 μM of genistein, 10 µM of EIPA, and 4 µM of cytochalasin D) and then added to the medium. After 24 and 72 h of incubation, the medium was replaced with a fresh medium containing 5 mg/mL of MTT and the cells were incubated for 3 h. Next, the cells were washed two times with phosphate-buffered saline (PBS) to remove detached dead cells and the formazan crystals were solubilized in DMSO. The absorbance was measured using a microplate reader at 570 nm. The relative cell viability (%) compared to non-treated cells was calculated by [abs] sample/[abs] control × 100.

### Transfection efficiency assay

HL-1 cells were seeded in 96-well plates and incubated to achieve 80% confluence 24 h prior to transfection. The medium was then replaced by a solution containing 1 µg/mL of HAp and 0.075, 0.3, or 0.75 µg of pEGFP dissolved in fresh medium. After 24 h, HL-1 cells were exposed to HAp/pDNA complexes and cultured for 24 h. HL-1 cells were stained by using the nuclear dye Hoechst 33343 (Dojindo) in the medium. The GFP expression was verified using a fluorescence microscope (20× magnification) and the transfection efficiency was defined as the percentage of EGFP-expressing cells. This percentage was calculated by dividing the number of EGFP-expressing cells by the total cell number. Data analysis was performed using MetaMorph software.

### Endocytic pathway assay

Endocytosis mechanisms were inhibited by treating the cells with pharmacological inhibitors and incubating them at 4 °C. HL-1 cells were pretreated using different inhibitors, such as 10 μM of chlorpromazine (Sigma) to inhibit clathrin-mediated endocytosis, 20 μM of genistein (Sigma) to inhibit caveolae-mediated endocytosis, 10 µM of EIPA (Cayman Chemical) and 4 µM of cytochalasin D (Sigma) to inhibit macropinocytosis. The cells were incubated for 30 min at 37 °C before the exposure to HAp/pDNA complexes. Energy-dependent endocytic processes were reduced by preincubating the cells at 4 °C for 30 min prior to HAp/pDNA complex exposure. After preincubation, the complexes (final concentration of 100 ng/mL) were added and incubated for 4 h, either in the presence of the inhibitors or at 4 °C. Then, the cells were washed three times using PBS to remove the remaining HAp nanoparticles and fresh medium was added. The cells were incubated for an additional 24 h before assessment. The relative transfection rate was normalized to that observed in the absence of inhibitors (100%).

### Macropinocytosis activity assay

HL-1 cells were seeded onto 35 mm glass-bottom culture dishes (Iwaki) and allowed to adhere for 24 h. The cells were preincubated in the presence or absence of 1 µg/mL of HAp nanoparticles for 1, 2, and 4 h. The cells were then incubated with 25 µg/mL of TMR–dextran (*M*_w_: 70,000) (Invitrogen) at 37 °C for 30 min. The cells were washed three times with PBS, to remove free TMR–dextran or membrane-bound dextran, fixed with 4% paraformaldehyde for 10 min, and stained with the nuclear dye Hoechst 33343. To quantify macropinocytosis activity, fluorescence microscopy images were obtained with BZ-70X (KEYENCE) using a magnification and exposure time similar to those used in previously published studies [40]. The macropinocytosis activity (relative fluorescence intensity per cell, *N* = 25) was calculated by using BZ Analyzer software.

### Statistical analysis

All the values are presented as the mean ± standard deviation (SD). The mean values were compared using the two-tailed Student’s *t*-test or one-way analysis of variance (ANOVA) followed by multiple comparisons using Ryan’s test. A value of *p* < 0.05 was considered to be statistically significant (ANOVA4 on the Web, Hiroshima Jogakuin University).
